# Exploring the relationship between lactate metabolism and immunological function in colorectal cancer through genes identification and analysis

**DOI:** 10.3389/fcell.2023.1173803

**Published:** 2023-08-24

**Authors:** Enkui Zhang, Xueliang Zhou, Xiaodong Fan, Shuchun Li, Chengsheng Ding, Hiju Hong, Batuer Aikemu, Guang Yang, Galiya Yesseyeva, Xiao Yang, Junjun Ma, Minhua Zheng

**Affiliations:** ^1^ Department of General Surgery, Ruijin Hospital, School of Medicine, Shanghai Jiao Tong University, Shanghai, China; ^2^ Shanghai Minimally Invasive Surgery Center, Ruijin Hospital, School of Medicine, Shanghai Jiao Tong University, Shanghai, China; ^3^ Department of General Surgery, Peking University First Hospital, Beijing, China; ^4^ Department of General Surgery and Carson International Cancer Research Center, Shenzhen University General Hospital and Shenzhen University Clinical Medical Academy, Shenzhen, China

**Keywords:** colorectal cancer, lactate metabolism, immune infiltration, prognosis, validation

## Abstract

**Introduction:** Metabolic dysregulation is a widely acknowledged contributor for the development and tumorigenesis of colorectal cancer (CRC), highlighting the need for reliable prognostic biomarkers in this malignancy.

**Methods:** Herein, we identified key genes relevant to CRC metabolism through a comprehensive analysis of lactate metabolism-related genes from GSEA MsigDB, employing univariate Cox regression analysis and random forest algorithms. Clinical prognostic analysis was performed following identification of three key genes, and consistent clustering enabled the classification of public datasets into three patterns with significant prognostic differences. The molecular pathways and tumor microenvironment (TME) of these patterns were then investigated through correlation analyses. Quantitative PCR was employed to quantify the mRNA expression levels of the three pivotal genes in CRC tissue. Single-cell RNA sequencing data and fluorescent multiplex immunohistochemistry were utilized to analyze relevant T cells and validate the correlation between key genes and CD4^+^ T cells.

**Results:** Our analysis revealed that MPC1, COQ2, and ADAMTS13 significantly stratify the cohort into three patterns with distinct prognoses. Additionally, the immune infiltration and molecular pathways were significantly different for each pattern. Among the key genes, MPC1 and COQ2 were positively associated with good prognosis, whereas ADAMTS13 was negatively associated with good prognosis. Single-cell RNA sequencing (scRNA-seq) data illustrated that the relationship between three key genes and T cells, which was further confirmed by the results of fluorescent multiplex immunohistochemistry demonstrating a positive correlation between MPC1 and COQ2 with CD4^+^ T cells and a negative correlation between ADAMTS13 and CD4^+^ T cells.

**Discussion:** These findings suggest that the three key lactate metabolism genes, MPC1, COQ2, and ADAMTS13, may serve as effective prognostic biomarkers and support the link between lactate metabolism and the immune microenvironment in CRC.

## Introduction

CRC remains the most prevalent cancer of the digestive system. Comprehensive treatment, mainly surgery, is the current mainstream approach to CRC treatment, with targeted therapies and immunotherapy being developed as important cutting-edge research (Dekker et al., 2019; Sung et al., 2021). While therapeutic options have led to improvements in overall survival of CRC patients, challenges persist in accurately predicting clinical prognosis and the likelihood of immunotherapy response (Lech et al., 2016). Given these challenges, there is an increasing interest among investigators to identify new biomarkers and elucidate tumor biological processes that could enhance the prediction of prognosis and identify relevant targets in CRC.

The TME comprises various components, including malignant cells, immune infiltrating cells, blood vessels, fibroblasts, and the extracellular matrix, that contribute greatly to tumorigenesis and tumor development (Quail and Joyce, 2013). Tumor cells interact with the TME is a critical aspect of invasion, metastasis, resistance to treatment, and other processes (Binnewies et al., 2018). Immune cells particularly play a crucial role in the TME, influencing targeted therapy, immunotherapeutic response, and survival prediction (Gajewski et al., 2013). The metabolic changes that occur within immune cells upon migration to the TME are also significant. Different immune cell subpopulations have distinct nutritional requirements for metabolic programming, and they can participate in various metabolic processes within the TME, impacting tumor progression (Dey et al., 2021). For instance, immune checkpoint inhibitors (ICIs) targeting programmed cell death protein 1 (PD-1), programmed cell death-Ligand 1 (PD-L1), and cytotoxic T lymphocyte-associated antigen-4 (CTLA-4), can reverse the tumor-induced metabolic restriction of T cell glucose, leading to the restoration of anti-tumor effects (Kraehenbuehl et al., 2022). Moreover, inherent differences in glutamine metabolism dependence are observed among different subtypes of macrophages, such as M1 and M2, which can be differentially utilized by cancer and immune cells for glutamine (Cruzat et al., 2018; Shang et al., 2020).

The “Warburg effect” is an important metabolic feature of tumors, and research on metabolic reprogramming related to tumor genesis and development is becoming one of the most cutting-edge research areas in oncology (Koppenol et al., 2011). In hypoxic conditions, cancer cells enhance their glycolytic activity leading to lactic acid accumulation in TME, which is then metabolized by neighboring cells, promoting metabolic reprogramming (Wang et al., 2020; Apostolova and Pearce, 2022). Lactic acid accumulation contributes to tumor cell proliferation, reduces TME pH, and inhibits the effectiveness of immune cells, leading to immunosuppression (Ippolito et al., 2019). Therefore, research on targeted lactate metabolism inhibition and lactate metabolism genes has become an important direction for cancer therapy. Different tumor metabolic patterns have distinct outcomes for CRC prognosis, and metabolic-related patterns and genetic markers show promising prognostic and predictive value (Xia et al., 2021; Lian et al., 2022). Thus, they hold potential to overcome the limitations of current models such as clinical TNM staging in accurately predicting tumor recurrence, metastasis, and survival in CRC patients.

**FIGURE 1:**
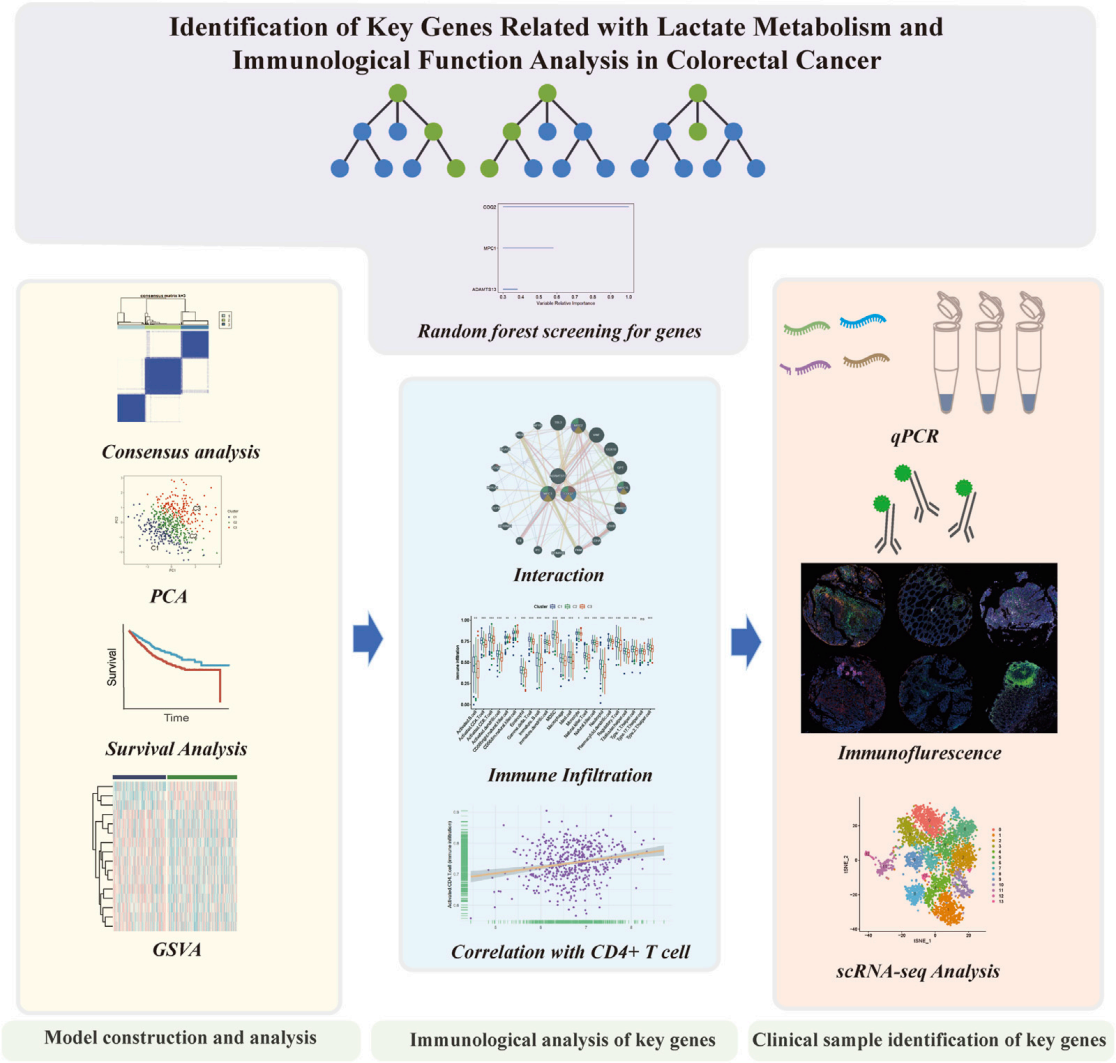
The flowchart of this study.

In our investigation, we examined the dissimilar expression of genes involved in lactate metabolism in CRC. Through bioinformatic methods, we identified significant lactate metabolism genes and evaluated the potential functions for predicting patient survival by dividing the CRC cohorts into three patterns. Then we analyzed the prospective molecular mechanisms and the effects on TME in the three clusters. Additionally, we conducted a comprehensive analysis of immune cell infiltration and clinical sample validation for the significant genes. Finally, we confirmed the role of these genes in the TME using scRNA-seq analysis (Figure 1).

## Materials and methods

### Public datasets collection

Human mRNA expression profiles and clinical information for CRC and normal adjacent tissues were downloaded from The Cancer Genome Atlas (TCGA). (https://tcga-data.nci.nih.gov/tcga/), and GSE39582 (Marisa et al., 2013) and GSE161158 (Szeglin et al., 2022) from NCBI Gene Expression Omnibus (GEO) databases (https://www.ncbi.nlm.nih.gov/geo/). The RNA-sequencing data originally provided as fragments per kilobase of transcript per million mapped reads (FPKM) values were converted to transcripts per million (TPM) values for analysis in the TCGA datasets. Then, we utilized the R package “limma” to download and normalized matrix files for microarray data from GEO. The present study employed a data integration strategy, specifically merging two distinct datasets (GSE39582 and GSE161158), followed by the implementation of the “Combet” algorithm using the “SVA” R package. The aim of this methodological approach was to effectively mitigate the impact of batch effects and thereby facilitate accurate and reliable downstream analyses (Muller et al., 2016).

### Cox regression analysis

We utilized the Cox proportional hazards model to conduct a univariate regression analysis in order to identify genes with a statistically significant *p*-value of less than 0.05. Specifically, the Cox regression analysis was employed as a means of narrowing down the pool of potential prognostic genes under investigation.

### Random forest

Applying random forest to downscale survival data of prognostic genes after Cox regression, ranked and filtered key genes according to variable importance. To perform feature selection, we leveraged the “randomForestSRC” package in this study. Specifically, we utilized a relative importance threshold of less than 0.4 to determine the final set of genes selected for analysis. The selected genes were subsequently ranked based on their importance scores, as determined by the random forest algorithm.

### Consensus clustering analysis

Consensus unsupervised cluster analysis was conducted to distribute CRC samples into distinct clusters, based on key gene expression profiles obtained from TCGA and GEO databases. To ensure consistent and reliable clustering, we employed the “ConsensusClusterPlus” R package. The optimal number of subgroups was determined through a combination of cumulative distribution function and consensus matrices analysis (Wilkerson and Hayes, 2010). Furthermore, so as to validate the clustering results obtained through the consensus unsupervised cluster analysis, Principal Component Analysis (PCA) was used as the clustering algorithm.

### GSVA and ssGSEA

To evaluate different biological processes for different patterns, we performed the non-parametric unsupervised analysis method of Gene Set Variation Analysis (GSVA). This involved converting the expression matrix of genes across samples into the expression matrix of gene sets across samples, thus enabling us to identify and compare differential pathways in different models. To conduct the GSVA analysis on CRC samples, we utilized the “GSVA” R package and the reference gene set “c2.cp.kegg.v7.5.1.symbols.gmt” obtained from the MSigDB database (Hanzelmann et al., 2013). In order to evaluate the infiltration level for immune cells in different patterns, we employed single-sample gene set enrichment analysis (ssGSEA) to calculate the relative abundance of immune cells in the TME. The immune cell gene sets used in this analysis were determined from previous studies (Charoentong et al., 2017).

### Correlation of lactate-related genes with clinical characteristics and prognosis in colorectal cancer patients

In addition, we evaluated different overall survival (OS) among the three patterns identified in our analysis. This was accomplished through the use of Kaplan-Meier analysis by utilizing the “Survival” and “Survminer” R packages.

### Fluorescent multiplex immunohistochemistry

Tissue microarray (TMA) slides were subjected to the following procedures: first, dewaxing with xylene, followed by rehydration through a graded ethanol series. Endogenous peroxidase activity was blocked using 3% hydrogen peroxide (Sinopharm Chemical Reagent Co., China, #73113760) for 10 min. The slides were then rinsed in PBS, underwent hot antigen repairing, and were subsequently washed with PBS. Next, 5% BSA (Sigma, Shanghai, China, # B2064) was added to each slide and incubated at room temperature for 20 min. Primary antibodies were then added to the slides in a volume of 100 μL each: COQ2 (Sino Biological, Rabbit anti-human mAb, 1:100, 206810-T08), MPC1 (SAB, Rabbit anti-human mAb, 1:200, #42898), and ADAMTS13 (SAB, Rabbit anti-human mAb, 1:150, #49953). The slides were incubated overnight at 4°C. The following day, the slides were rinsed with PBS and incubated with a labeled secondary antibody (Abcam, Goat anti-rabbit IgG H&L (HRP), 1:2000, ab205718) at 37°C for 30 min. After rinsing again with PBS, each section was treated with 100ul try-488 Tyramine Conversion Reagent (runnerbio, Bry-try488) and incubated for 10–30 min at room temperature. Finally, the sections were mounted with anti-fluorescence quenching sealer containing DAPI (Beyotime Biotechnology, China, P0131).

### Real time quantitative PCR (RT-qPCR)

The primers utilized for qPCR analysis were designed through the use of Primer 6.0 software. The primer sequences of all genes were listed as follows: COQ2-F: GGG​GAG​CGT​TAC​TTG​GAT​GG, COQ2-R: AAC​CGC​AGA​GCC​GTT​GAC​TT; MPC1-F: CCC​TCT​GTT​GCT​ATT​CTT​TGA​C, MPC1-R: TAC​TTC​ATT​TGT​TGC​GTG​GC; ADAMTS13-F: TGG​TCG​TGT​CGA​GTA​CAG​AGT​G, ADAMTS13-R: CGT​GGC​TTA​GGC​TGG​AAG​TA. RT-qPCR analyses were quantified with SYBR-Green (BioTNT, Shanghai, China), and the levels were normalized to the level of ACTB.

### Single cell RNA-seq (scRNA-seq) data process

The CRC scRNA-seq dataset GSE132257 (Lee et al., 2020) was downloaded as required from the GEO database. Two colorectal cancer (CRC) patients underwent scRNA-seq analysis on cancer or distant normal tissue dissociates. The scRNA-seq data in Seurat object format, containing gene expression information, was imported into the Seurat (v2.3.0) R toolkit using the Read10× () function (Satija et al., 2015). A total of 18,409 cells from 10 sample preparations were analyzed. The integrated data was normalized by scaling, followed by the t-Distributed stochastic neighbor embedding (tSNE) method. Annotating single cell data was performed using the “celldex” R package. Further, the T-cell subpopulations were annotated using the “HumanPrimaryCellAtlasData_fine” dataset. Additionally, to estimate cell differentiation, we employed analysis via CytoTRACE, which is a validated method for forecasting cell differentiation using scRNA-seq data (Gulati et al., 2020).

### Statistical analysis

Statistical analysis of the data was carried out using R-4.1.2 and GraphPad Prism 9. For normally distributed variables, statistical significance was evaluated using the Student's t-test, while for nonparametric or parametric methods, Wilcoxon test and Kruskal-Wallis test were used (Hazra and Gogtay, 2016). It was decided to conduct all statistical analyses on a two-sided basis, with a probability level of 0.05 being considered statistically significant.

## Results

### Characteristics and screening of key genes involved in lactate metabolism

Characterizing lactate metabolism genes and screening key genes associated with HP_INCREASED_SERUM_LACTATE were obtained from GSEA MSigDB, 195 lactate metabolism genes were collected for comprehensive analysis (Supplementary Table S1). Differential gene expression analysis was conducted to compare the expression of genes involved in lactate metabolism between normal and tumor tissues. The differentially expressed genes were visualized in CRC samples obtained from TCGA database (Supplementary Figure S1A). Most lactate metabolism genes were highly expressed in tumor tissues, and we further analyzed the protein-protein interactions of the differential genes (Supplementary Figure S1B). A univariate Cox regression analysis was conducted on 195 genes involved in lactate metabolism in a cohort of 515 patients with CRC obtained in the TCGA dataset. This analysis identified 15 genes that were found to be prognostic and are presented in a forest plot (Supplementary Figure S1C; Supplementary Table S2). Gene ontology and KEGG pathway analyses were also applied on lactate metabolism genes (Supplementary Figure S2A, B).

**FIGURE 2:**
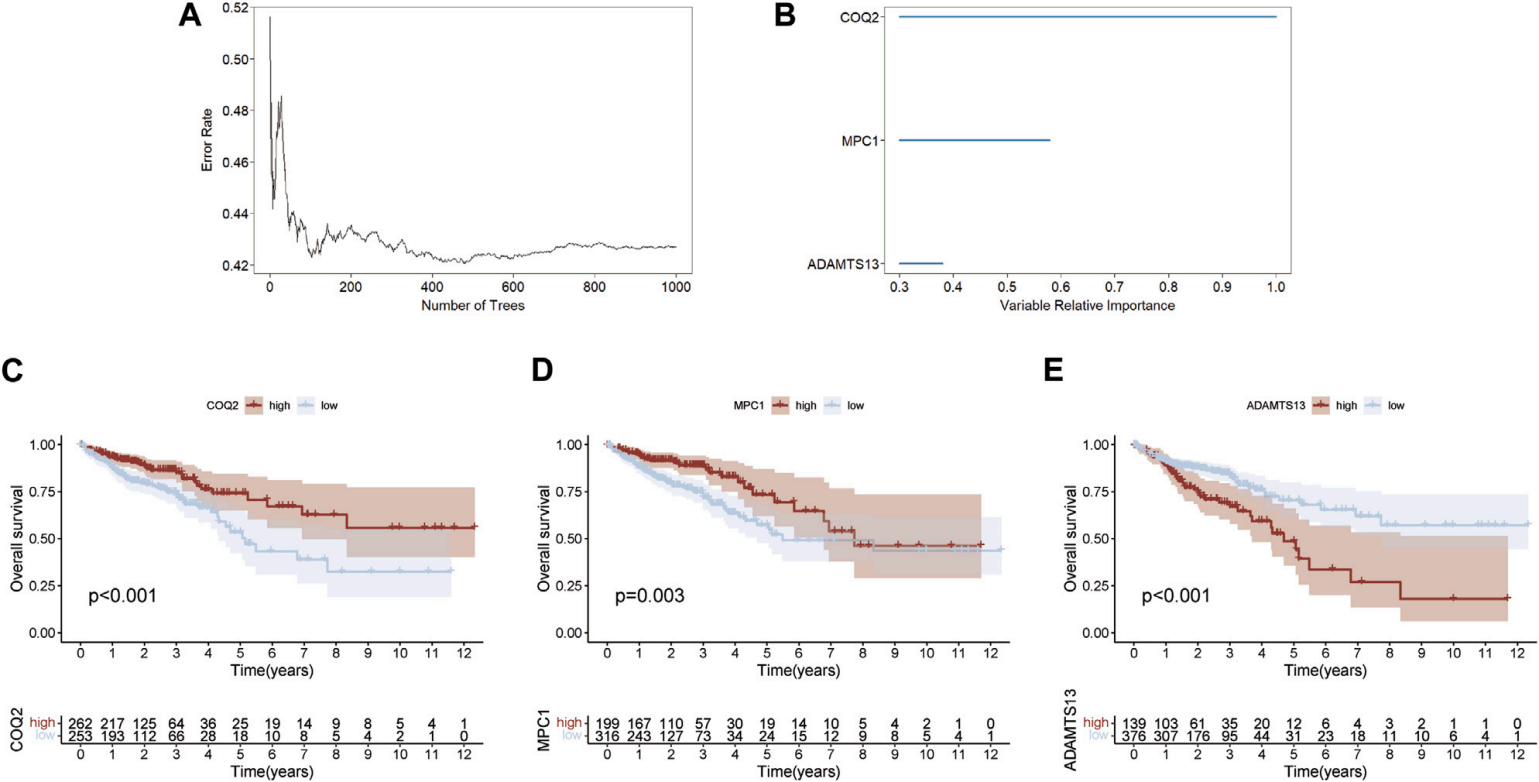
Random forest screening for lactate metabolism genes **(A)** Error rate for the data as a function of the classification tree. **(B)** Random forest was used to identify key genes in the prognostic genes (relative importance threshold <0.4). **(C–E)** Overall survival prognosis and survival curves of COQ2, MPC1, ADAMTS13 for CRC samples.

A random forest method was employed to select key genes from 15 prognostic genes, based on their relative importance scores using the “randomForestSRC” package for further selection. Genes with a score <0.4 were excluded, and the top three genes on variable importance were identified as COQ2, MPC1, and ADAMTS13 (Figures 2A, B). The association between the number of classification trees and the error rate was examined (Supplementary Table S3). Using the expression levels of these three key genes, we predicted the prognosis of CRC patients and found that high expression of COQ2 and MPC1 was associated with better prognosis, while high expression of ADAMTS13 corresponded to a negative prognosis (Figures 2C–E). These results suggest that the expression levels of COQ2, MPC1, and ADAMTS13 have the potential to serve as prognostic biomarkers for CRC.

### Molecular types based on lactate metabolism key genes

**FIGURE 3:**
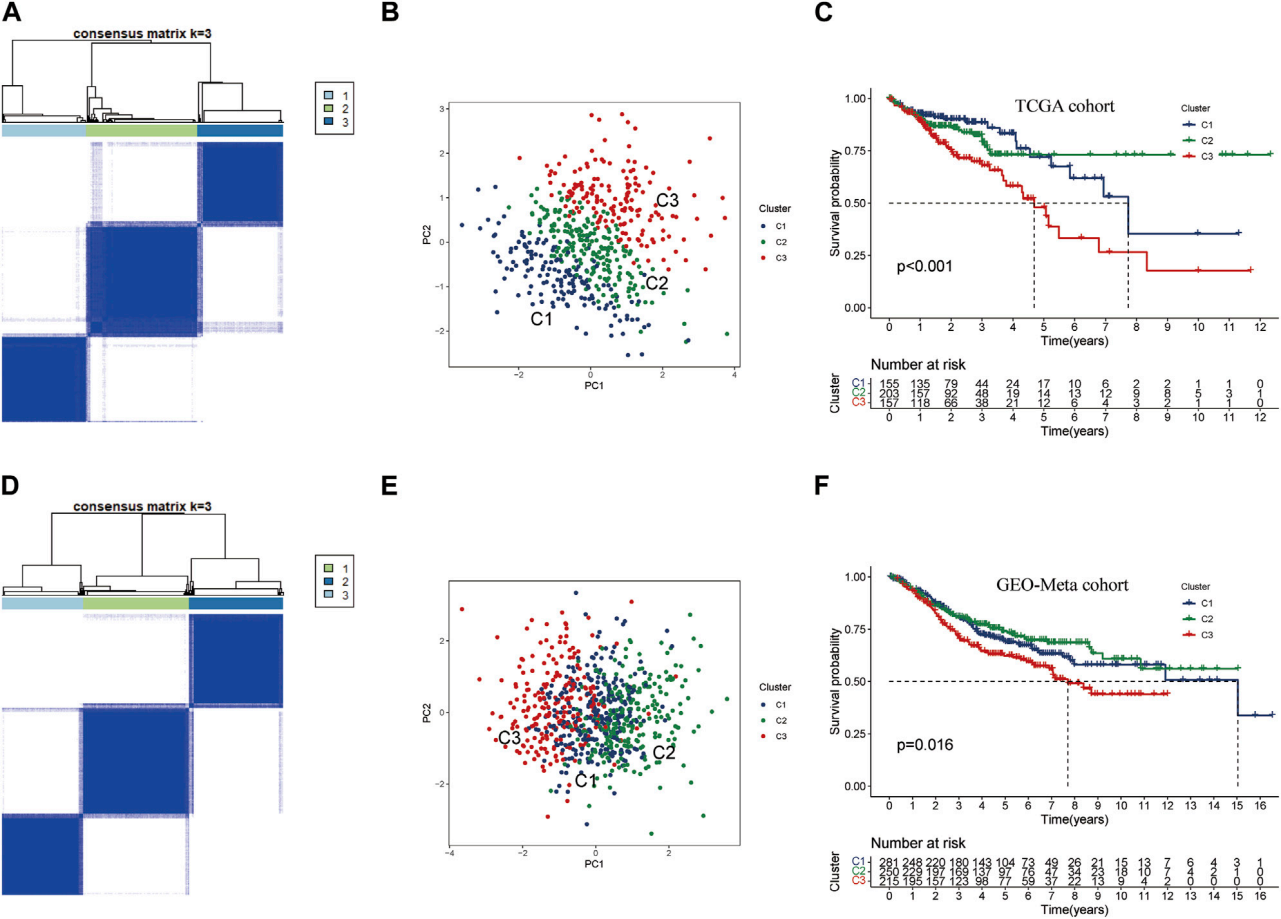
Consensus cluster analysis of key genes in lactate metabolism **(A)** CRC samples from TCGA were used to construct a prognostic signature. The cohort was divided into three patterns when *k* = 3. **(B)** PCA algorithm divided CRC samples from TCGA into three clusters. **(C)** Overall survival rates for Cluster C3 were significantly lower than those for Cluster C1 and Cluster 2. p < 0.05. **(D)** To build the prognostic signature for CRC samples from GEO-Meta cohort (GES39582, GES161158) by consensus clustering. The cohort was also divided into three patterns when *k* = 3. **(E)** In GEO-Meta, three clusters of CRC samples were determined using the PCA algorithm. **(F)** In GEO-Meta, Cluster C3 also had the shortest overall survival rate compared to Cluster C1 and Cluster 2. p < 0.05.

To investigate the potential clinical value and underlying mechanisms of lactate metabolism key genes in CRC, we utilized the TCGA database to perform consistent clustering analysis on 515 samples (Figure 3A). Using the cumulative distribution function plots and tracking plot, we classified the TCGA-CRC cohort into three distinct patterns based on the expression levels of the three key genes. Furthermore, we employed PCA on the TCGA cohort and found that the three clusters were well-separated (Figure 3B; Supplementary Table S4). Notably, Kaplan-Meier survival analysis indicated CRC patients in Cluster C3 had the worst prognosis, while those in Cluster C1 and Cluster C2 had better prognoses (Figure 3C). To validate these results, we conducted consistent clustering and PCA analysis on an additional set of 746 CRC samples from the GEO-meta cohort by merging GSE39582 and GSE161158 datasets (Supplementary Table S4). The clustering results of the validation set were consistent with those of the TCGA cohort, successfully separating the samples into three clusters (Figures 3D, E). Consistent with the TCGA results, the survival analysis of the GEO-meta cohort demonstrated that Cluster C3 was associated with the shortest prognosis, while Cluster C1 and C2 were associated with longer prognoses (Figure 3F).

### Analysis of different pathways and clinical features in subtypes

To explore the characteristics of the clustering model constructed by lactate metabolism key genes, we analyzed and summarized their associated differential pathways and clinical features.

**FIGURE 4:**
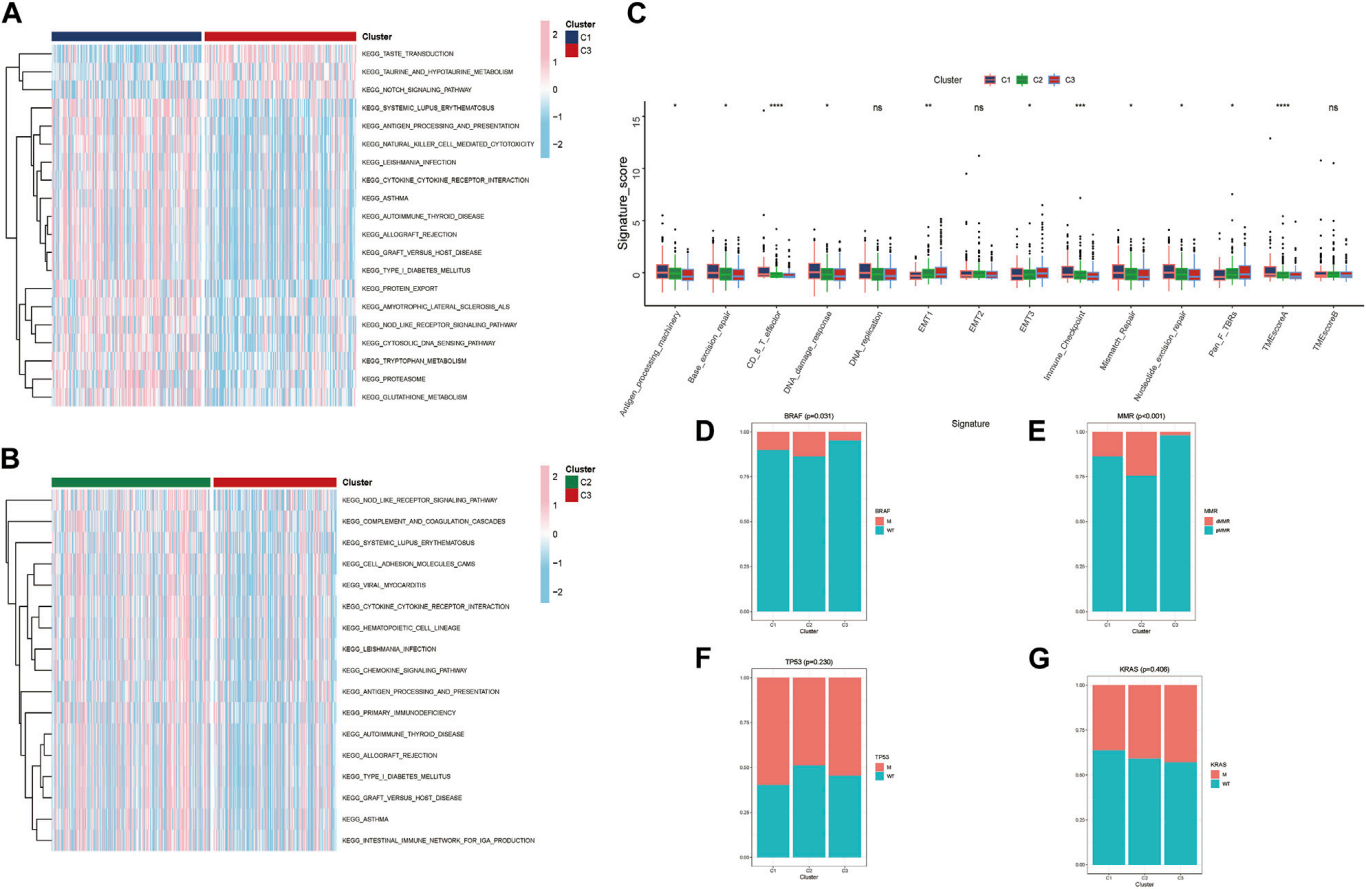
Molecular features of different clusters **(A, B)** Three distinct clusters of KEGG biological pathways are identified by GSVA enrichment analysis; pink represents active pathways, while blue represents inhibited pathways. **(C)** Boxplot showing each TME signature for each cluster in the TCGA cohort. **(D–G)** Based on GES39582, the proportion of mutation features in each cluster. (* *p* < 0.05; ** *p* < 0.01; *** *p* < 0.001; **** *p* < 0.0001).

In the TCGA-CRC cohort, GAVA was displayed to analyze the different pathways within clusters. We found that part of pathways about immune defense and cytokine/receptor interaction had lower expression in Cluster C3 verse Cluster C2 and C1. These immune related signal pathways mainly included ANTIGEN PROCESSING AND PRESENTATION, PRIMARY IMMUNOEFICIRNCY, CHEMOKINE SIGNALING PATHWAY and so on (Figures 4A, B). The results indicated that the poor prognosis of Cluster C3 in the clustering pattern corresponding to lactate metabolism key genes had close relationship with the lower expression of immune signaling pathways. Moreover, TME-related biological signatures (Zeng et al., 2019) had been evaluated the different clusters characteristics (Figure 4C). The results revealed that the oncogenic signatures of the TME associated with poorer prognosis Cluster C3 were generally higher, including EMT1, EMT3, and Pan-fibroblast TGF-β response signature (Pan-F-TBRs). Conversely, the TME anti-cancer signatures corresponding to better prognosis Clusters C1 and C2 were stronger, such as Antigen-processing-machinery, Genetic-repair-signature, CD8^+^ T effector, DNA-damage-response, Immune-checkpoint, and TMEscoreA. Furthermore, we also analyzed the differences in clinical features among the three clusters from GES39582 and found that BRAF and MMR had significant variations. The minimum number of mutations for BRAF and MMR were observed in the poor prognosis Cluster C3 (Figures 4D–G). Moreover, we conducted a correlation analysis of the proteins corresponding to the three key genes. GeneMANIA database analysis indicated that these three proteins interacted with several metabolism-related proteins and might be involved in processes related to lactate metabolism (Supplementary Figure S3A). Furthermore, we utilized the STITCH database to demonstrate drugs with *p* value < 0.05 and investigate the proteins targeted by corresponding drugs to elucidate the possible targets and signal pathways associated with the three proteins. (Supplementary Figures S3B–D).

### Relationship between lactate metabolism key genes and tumor immune microenvironment in CRC

**FIGURE 5:**
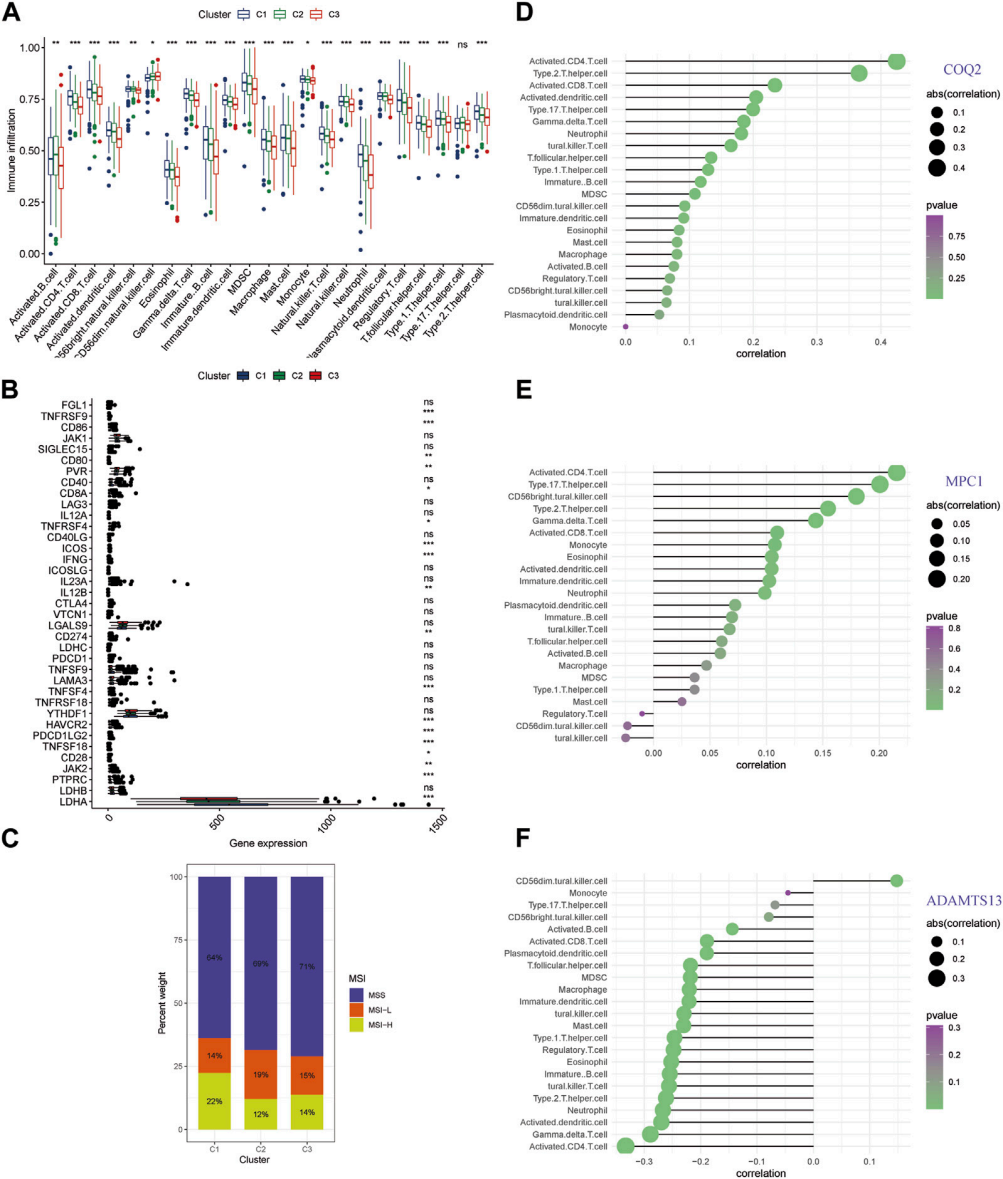
Immune infiltration analysis of lactate metabolism gene clusters **(A)** An analysis of the RNA-seq meta cohort shows that immune cells are abundant in three patterns. **(B)** Comparing gene expression of major immune checkpoints among three clusters using boxplots. **(C)** Microsatellite instability in the three clusters is represented by the proportion of patients. **(D–F)** The three key genes are correlated with immune infiltration. (* *p* < 0.05; ** *p* < 0.01; *** *p* < 0.001).

Distinct pathways indicating an immune-related influence have been confirmed across different clusters. To further explore this direction, we examined immune infiltrating and the genes of immune checkpoint in the clusters (Supplementary Table S5). Cluster C3 exhibited lower levels of conventional anti-tumor immune cells, including activated B cells, CD8^+^ T cells, CD4^+^ T cells, and NK cells, as compared with C1 and C2 (Figure 5A). Moreover, we conducted correlation analysis for immune checkpoint expression level for the different patterns, and found that C3 had lower expression levels of the several genes including TNFRSF9, CD86, CD80, PVR, CD8A, TNFRSF4, ICOS, IFNG, IL12B, CD274, TNFSF4, HAVCR2, PDCD1LG2, TNFSF18, CD28, JAK2, PTPRC, and LDHA (Figure 5B). C3 had the highest percentage of MSS among the clusters, which was associated with a poorer prognosis, whereas C2 and C1 had the highest percentage of MSI-H and were associated with a better prognosis (Figure 5C). These findings suggest that the three key genes involved in lactate metabolism may be closely linked to the immune microenvironment and immunotherapy. To further investigate this relationship, we performed ssGSEA analysis of immune infiltration for the three genes (Figures 5D–F). The results indicated that COQ2 and MPC1 were positively correlated with the majority of immune cells, while ADAMTS13 was negatively correlated with the majority of immune cells. Notably, CD4^+^ T cells were found to be strongly associated with all three genes.

### Validation of the key genes with clinical samples and scRNA-seq analysis

**FIGURE 6:**
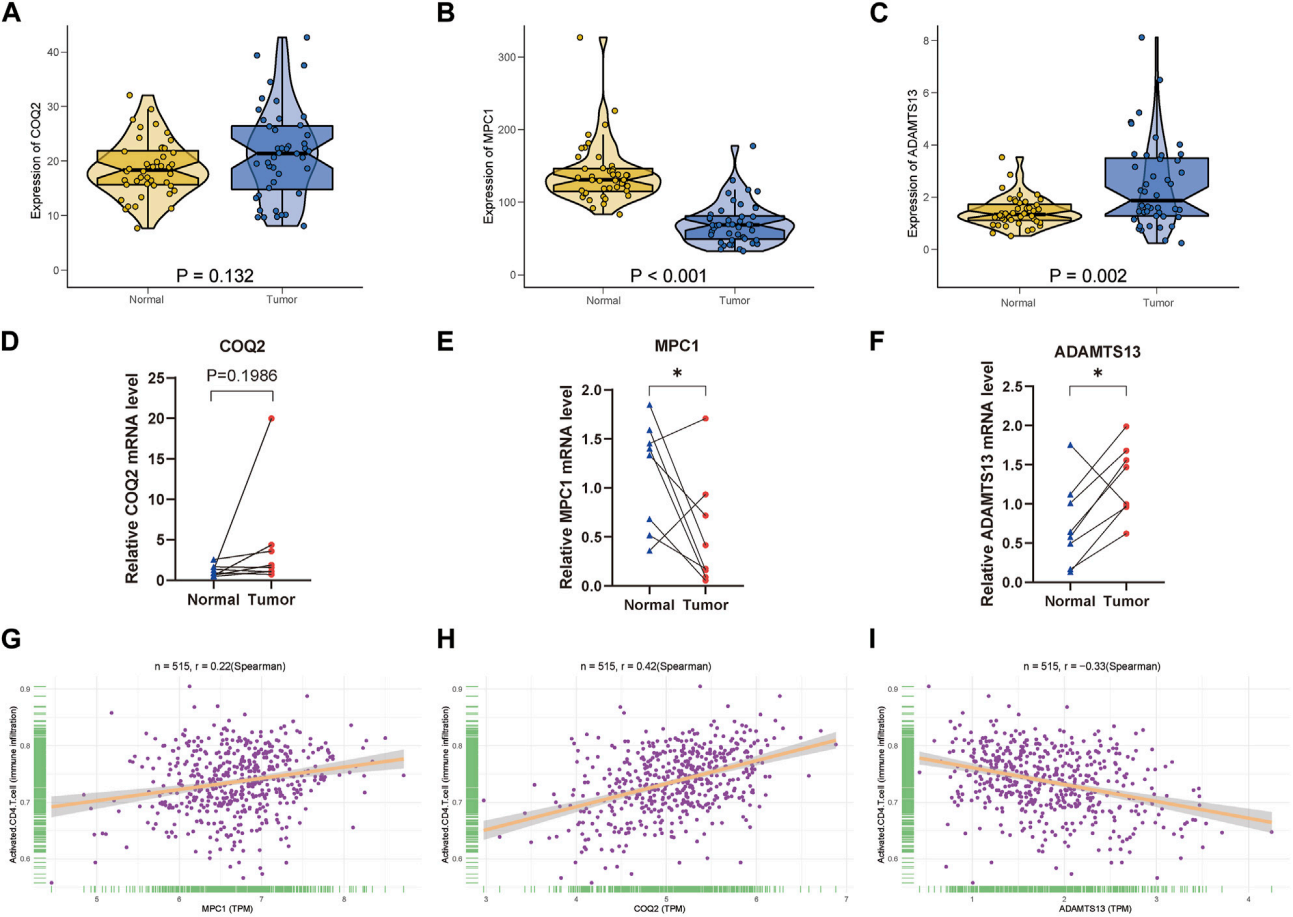
Clinical verification of key genes and correlation analysis of CD4^+^ T cells **(A–C)** Expression of COQ2, MPC1, ADAMTS13 between normal and tumor tissues in TCGA CRC samples. **(D–F)** According to the qPCR results, three key genes are expressed in CRC samples, *n* = 8. **(G–I)** Relative plot of activated CD4^+^ T cell with the three key genes. (* *p* < 0.05; ** *p* < 0.01; *** *p* < 0.001).

**FIGURE 7:**
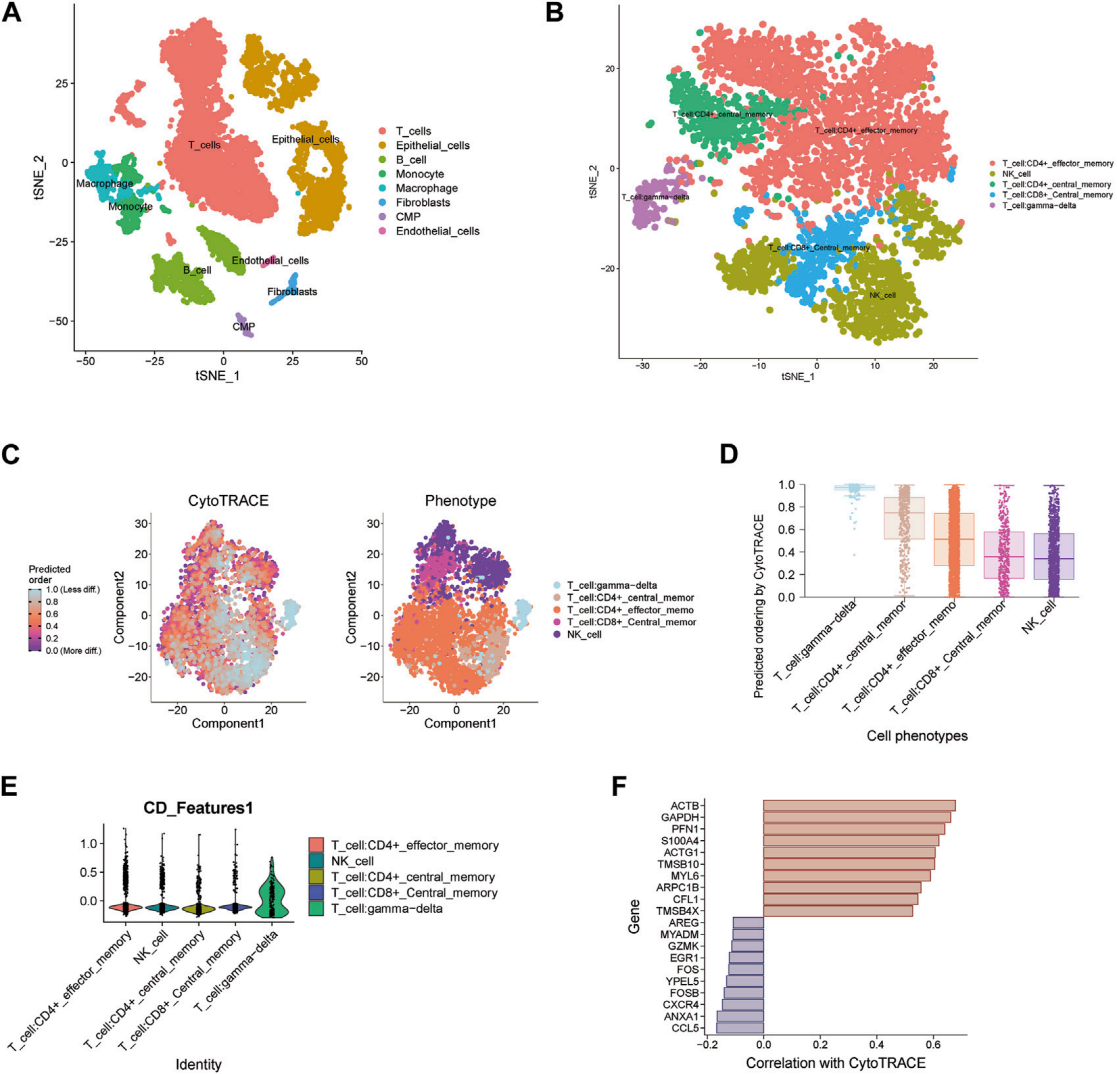
Single-cell data analysis of key genes **(A)** t-SNE plot of 8 cell clusters in GSE132257. **(B)** Cell distribution in T cell cluster. **(C–D)** Using CytoTRACE, differentiation status of different T cell types is determined. A lower score indicates lower differentiation. **(E)** Expression of three key genes in different T cell subsets. **(F)** Correlation genes between T cells and lactate metabolism with CytoTRACE.

For the three key genes COQ2, MPC1 and ADAMTS13, we analyzed the gene expression in CRC and the adjacent tissues of corresponding patients in the TCGA database (Figures 6A–C). It was observed that the expression level of MPC1 was clearly elevated in adjacent normal tissues comparing with CRC, while ADAMTS13 was considerably upregulated in tumor tissues in contrast to normal tissues. However, there was no statistically significant differences observed in the expression levels of COQ2 in CRC and normal tissues. Afterwards, to confirm the expression of the key genes, we also collected the patient’s tumor tissue and the corresponding normal tissue from Ruijin Hospital and performed RT-qPCR (Figures 6D–F). The expression of the three genes, COQ2, MPC1, and ADAMTS13, in normal and tumor tissues was analyzed, revealing that the expression of MPC1 was higher in normal tissues, while ADAMTS13 was higher in tumor tissues. COQ2 expression did not differ significantly between the two types of tissues. Further analysis was performed to investigate the correlation between the expression of these genes and CD4^+^ T cells in TCGA samples (Figures 6G–I). The results showed a positive association between MPC1 and COQ2 with CD4^+^ T cells, while ADAMTS13 was negatively associated with CD4^+^ T cells. Based on our findings, we conducted a prognostic analysis focusing on the potential oncogene ADAMTS13. In the univariate Cox hazard analysis, ADAMTS13 emerged as a significant risk factor, demonstrating a strong predictive role in patients with colorectal cancer (CRC). However, its significance diminished to some extent in the multivariate analysis (Supplementary Figures S4A, B). To validate the accuracy of the nomogram, we performed a calibration analysis (Supplementary Figure S4C). Encouragingly, the results indicated that the predicted line in the nomogram closely approximated the actual survival rate (Supplementary Figure S4D). These findings suggest that ADAMTS13 might serve as a potential prognostic biomarker in CRC and warrant further investigation. We further conducted an analysis of the functional pathway correlations of ADAMTS13 with cancer development in CRC samples. The results revealed a significant association with various pathways, including tumor progression, cell migration and invasion abilities, tumor proliferation, and metabolism. These findings suggest that ADAMTS13 may play an active role in these pathways in the context of CRC development (Supplementary Figures S5A–I). These findings led to further investigation of the immune microenvironment. Using the t-SNE method, GSE132257 cells were grouped into 8 major cell types, including T cells, epithelial cells, B cells, monocytes, macrophages, fibroblasts, Common Myeloid Progenitors (CMP), and endothelial cells (Figure 7A). We found that multiple T cell subsets were closely associated with three key genes in immune infiltration analysis, so we further analyzed and annotated the T cell subsets, mainly five cell subtypes: CD4^+^ effector memory cell, NK cell, CD4^+^ central memory cell, CD8^+^ central memory cell and gamma-delta T cell (Figure 7B). Next, we compared the differentiation potential of different T-cell subtypes. We estimated a higher differentiation potential for gamma-delta T cell and CD4^+^ T cell based on CytoTRACE, (Figures 7C, D). We found that three key genes were most abundantly expressed in gamma-delta T cells (Figure 7E). Besides, we also showed the correlation genes between T cells and lactate metabolism with CytoTRACE (Figure 7F).

### Validation of the relationship between key genes with CD4^+^ T cell

**FIGURE 8:**
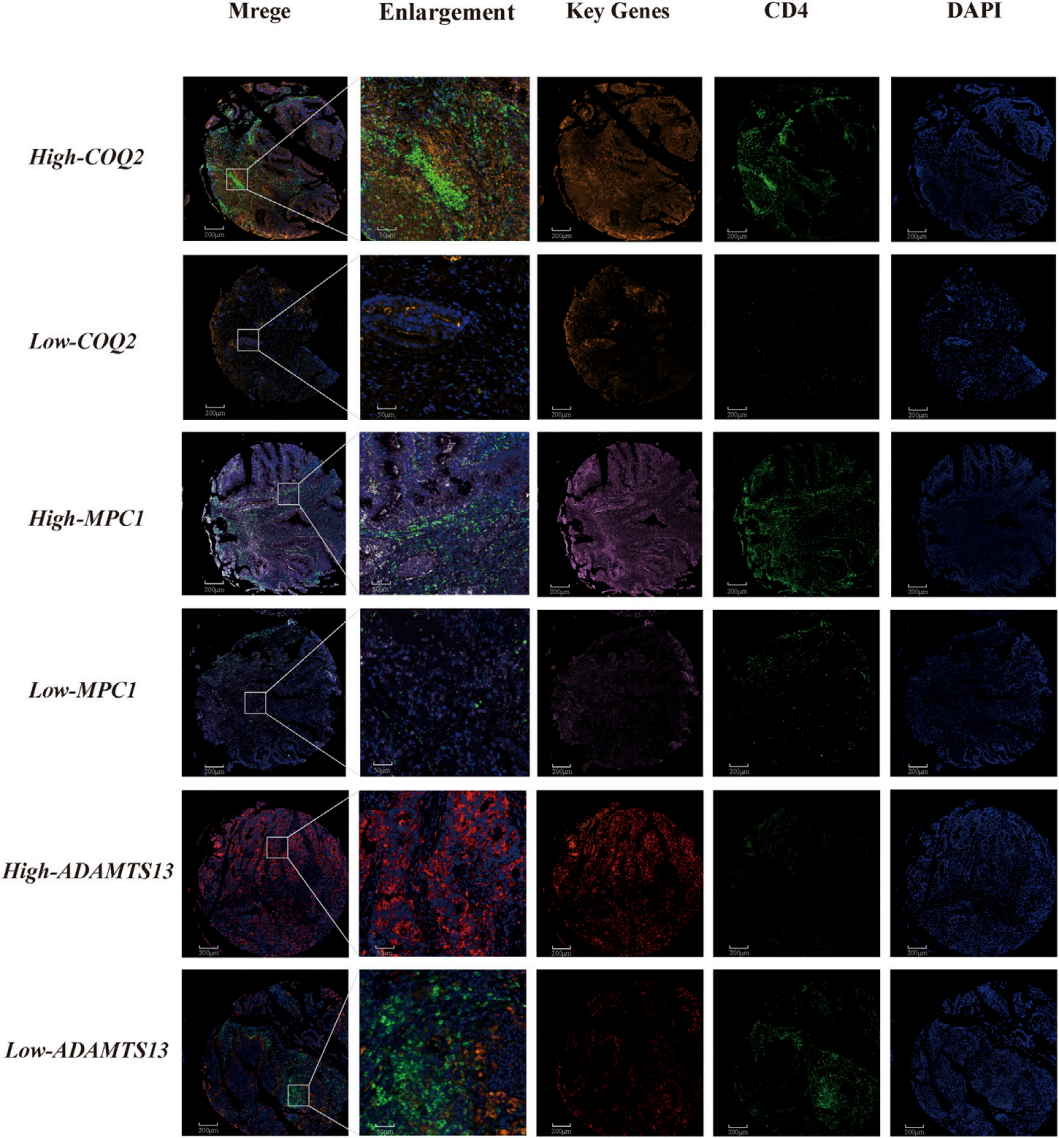
Tissue immunofluorescence identification of the relationship between CD4^+^ T cells and key genes in lactate metabolism Fluorescent multiplex immunohistochemistry for the relationship between COQ2, MPC1, ADAMTS13 and CD4^+^ T cell.

We further investigated the correlation between CD4^+^ T cells and three genes, namely, MPC1, COQ2, and ADAMTS13, in CRC patients. The results demonstrated a positive correlation between CD4^+^ T cells and MPC1 and COQ2, while a negative correlation between CD4^+^ T cells and ADAMTS13. Notably, the correlation between COQ2 and ADAMTS13 was found to be stronger than that between the other gene pairs. To gain a deeper understanding of the relationship between the relevant T cells and the three genes at the single cell level, further analysis was conducted. In addition, fluorescent multiplex immunohistochemistry was carried out in CRC tissue microarrays to examine the expression of the three key genes (Figure 8). Double-labeled fluorescence localization of CD4 and the three genes separately revealed that higher expression of COQ2 and MPC1 was related with higher levels of CD4 signaling, while low expression of COQ2 and MPC1 corresponded to lower levels of CD4 signaling. In contrast, high expression of ADAMTS13 was associated with lower levels of CD4 signaling. These findings provide further support for the initial observation that COQ2 and MPC1 are positively correlated with CD4^+^ T cells, while ADAMTS13 is negatively correlated with CD4^+^ T cells (Supplementary Figure S4).

## Discussion

CRC is a cancer with a high degree of heterogeneity and complex molecular mechanisms. Metabolic reprogramming has been identified as a pivotal regulator of tumorigenesis and progression in CRC (Faubert et al., 2020), and lactate and lactate-mediated signaling pathways have been found to contribute to various aspects of tumor progression (Brown and Ganapathy, 2020; Jin et al., 2021). As glucose metabolism increases dramatically and lactate accumulates in tumor cells during rapid tumor growth, lactate is considered a metabolic product that promotes cancer cell proliferation (Vaupel et al., 2019). Therefore, the identification of key molecules involved in lactate metabolism as new biological markers for the explanation of CRC molecular mechanisms and clinical prognosis holds significant promise. Additionally, the potential impact of the TME on tumor metabolism is substantial. As critical components of the TME, immune cells are likely to play a role in regulating lactate metabolism (Andrejeva and Rathmell, 2017; Madden and Rathmell, 2021). Therefore, exploring the interactions between metabolism and immunity is particularly important, as this could provide a reliable basis for investigating how metabolites affect tumor progression.

In the present study, COX regression and random forest methods were utilized to screen lactate metabolism genes in the CRC cohort, resulting in the identification of three key genes: MPC1, COQ2, and ADAMTS13. According to previous studies, decreased MPC1 expression results in increased glycolysis and compensatory glutamine metabolism (Grenell et al., 2019; Jiang et al., 2022). COQ2 is involved in the synthesis of CoQ10, which in turn affects the electron transport chain and aerobic respiration in cellular mitochondria. Decreased expression of COQ2 disrupts the normal process of the tricarboxylic acid cycle (Rabanal-Ruiz et al., 2021). Increased ADAMTS-13 activity is associated with increased serum lactate, an important plasma molecule in lactate metabolism that has been implicated in tumor progression, metastasis and micro thrombosis (Garam et al., 2018; Faqihi et al., 2021). The clinical prognostic ability of these three genes was validated in the TCGA cohort, where high expression of COQ2 and MPC1 was associated with better prognosis for CRC patients, while high expression of ADAMTS13 was associated with worse prognosis.

Using these findings, a consistent clustering method that incorporated the three key genes was developed, which successfully separated the TCGA and meta-GEO cohorts into three distinct patterns. These patterns exhibited significant differences in survival prognosis in both cohorts, and further molecular pathway and clinical feature analyses of the three patterns were consistent with the prognostic analysis. Additionally, Immune infiltration analysis is an important method for immune cell-related analysis of transcriptome data (Newman et al., 2015). Our results revealed that poor prognosis in one of the patterns was highly correlated with high levels of immune-suppressive cell infiltration, while good prognosis in the other two patterns was closely related to T cells and B cells. The consistency clustering model using the three key genes can effectively distinguish clinical patient prognosis and is useful for related basic research. Moreover, immune infiltration analysis and clinical sample validation were performed for each of the three genes, and it was depicted that all three genes were highly related with distinct types of T cells, especially CD4^+^ T cells being the most correlated immune cells. Besides, the expression of the three key genes was found to vary between tumor and normal tissues, with COQ2 showing a trend of higher expression in tumors, MPC1 being expressed more in normal tissues, and ADAMTS13 being expressed more in tumors. We also conducted explorations concerning the potential oncogene, ADAMTS13, among the key genes. We found that ADAMTS13, as a potential risk gene in tumors, could serve as a clinical prognostic marker for CRC. Moreover, ADAMTS13 exhibited close correlations with multiple pathways related to tumor occurrence, tumor cell proliferation, and migration. These findings suggest that ADAMTS13, as a crucial gene in lactate metabolism, may play a significant role in the development of CRC. As a result, further investigations into the role of this gene are warranted to gain deeper insights into its relevance and implications in the future.

Single-cell omics analysis has significant advantages for studying the relationship between immune cells and the TME (Zhang and Zhang, 2020). Based on our above findings regarding the close connection between lactate metabolism and the immune system, we further analyzed, through scRNA-seq and immunofluorescence histology validation, the potential relationship between three key genes and the T cells with the closest connection. In the single-cell data, gamma-delta T cells may play a larger role among the three genes. In the validation through immune fluorescence tissue analysis, we further validated the relationship between the three genes and CD4 in detail, providing a more comprehensive verification of our previous results.

The study found that in our model and gene verification results, MPC1, COQ2, and ADAMTS13 play important roles in the prognosis of CRC patients. We predict that these three key genes in lactate metabolism can affect the clinical prognosis of patients by influencing the immune microenvironment. T cells are considered to be the most profound immune cells affecting immune response in TME (Joyce and Fearon, 2015). Related studies on T cells and lactate metabolism are also underway (Kumagai et al., 2022). The findings of our study, based on multiple datasets, indicate that patients with elevated levels of MPC1 and COQ2 exhibit improved prognoses, whereas those with high levels of ADAMTS13 are associated with poorer prognoses. Furthermore, high expression of MPC1 and COQ2 corresponds to high expression of CD4, while high expression of ADAMTS13 corresponds to low expression of CD4. These findings suggest that the lactate key genes identified in this study may impact tumor function and patient outcomes by modulating T-cell subsets. However, the underlying communication mechanism involved in this process warrants further investigation in future studies.

## Conclusion

In general, we utilized random forest approach to screen for representative lactate metabolism genes and successfully employed this method to consistently cluster CRC patients, revealing significantly different patterns. We observed that immune cells in the TME, particularly T cells, were closely correlated with the expression of identified genes. The expression of these three key genes and their relationship with CD4^+^ T cells were further confirmed through qPCR and tissue immunofluorescence analysis. Our results indicated that these key genes had potential as novel prognostic markers for CRC. Additionally, we confirmed the potential role of lactate metabolism genes and immune cells in the TME, providing a basis for investigating the underlying mechanisms of lactate metabolism on TME and for conducting immunotherapy studies.

## Data Availability

The original contributions presented in the study are included in the article/Supplementary Material, further inquiries can be directed to the corresponding authors.

## References

[B1] Andrejeva G. Rathmell J. C. (2017). Similarities and distinctions of cancer and immune metabolism in inflammation and tumors. Cell. Metab. 26 (1), 49–70. 10.1016/j.cmet.2017.06.004 28683294 PMC5555084

[B2] Apostolova P. Pearce E. L. (2022). Lactic acid and lactate: revisiting the physiological roles in the tumor microenvironment. Trends Immunol. 43 (12), 969–977. 10.1016/j.it.2022.10.005 36319537 PMC10905416

[B3] Binnewies M. Roberts E. W. Kersten K. Chan V. Fearon D. F. Merad M. (2018). Understanding the tumor immune microenvironment (TIME) for effective therapy. Nat. Med. 24 (5), 541–550. 10.1038/s41591-018-0014-x 29686425 PMC5998822

[B4] Brown T. P. Ganapathy V. (2020). Lactate/GPR81 signaling and proton motive force in cancer: role in angiogenesis, immune escape, nutrition, and Warburg phenomenon. Pharmacol. Ther. 206, 107451. 10.1016/j.pharmthera.2019.107451 31836453

[B5] Charoentong P. Finotello F. Angelova M. Mayer C. Efremova M. Rieder D. (2017). Pan-cancer immunogenomic analyses reveal genotype-immunophenotype relationships and predictors of response to checkpoint blockade. Cell. Rep. 18 (1), 248–262. 10.1016/j.celrep.2016.12.019 28052254

[B6] Cruzat V. Macedo Rogero M. Noel Keane K. Curi R. Newsholme P. (2018). Glutamine: metabolism and immune function, supplementation and clinical translation. Nutrients 10 (11), 1564. 10.3390/nu10111564 30360490 PMC6266414

[B7] Dekker E. Tanis P. J. Vleugels J. L. A. Kasi P. M. Wallace M. B. (2019). Colorectal cancer. Lancet 394 (10207), 1467–1480. 10.1016/S0140-6736(19)32319-0 31631858

[B8] Dey P. Kimmelman A. C. DePinho R. A. (2021). Metabolic codependencies in the tumor microenvironment. Cancer Discov. 11 (5), 1067–1081. 10.1158/2159-8290.CD-20-1211 33504580 PMC8102306

[B9] Faqihi F. Alharthy A. Abdulaziz S. Balhamar A. Alomari A. AlAseri Z. (2021). Therapeutic plasma exchange in patients with life-threatening COVID-19: a randomised controlled clinical trial. Int. J. Antimicrob. Agents 57 (5), 106334. 10.1016/j.ijantimicag.2021.106334 33838224 PMC8024223

[B10] Faubert B. Solmonson A. DeBerardinis R. J. (2020). Metabolic reprogramming and cancer progression. Science 368 (6487), eaaw5473. 10.1126/science.aaw5473 32273439 PMC7227780

[B11] Gajewski T. F. Schreiber H. Fu Y. X. (2013). Innate and adaptive immune cells in the tumor microenvironment. Nat. Immunol. 14 (10), 1014–1022. 10.1038/ni.2703 24048123 PMC4118725

[B12] Garam N. Maláti É. Sinkovits G. Gombos T. Szederjesi A. Barabás L. (2018). Platelet count, ADAMTS13 activity, von Willebrand factor level and survival in patients with colorectal cancer: 5-Year follow-up study. Thromb. Haemost. 118 (1), 123–131. 10.1160/TH17-07-0548 29304532

[B13] Grenell A. Wang Y. Yam M. Swarup A. Dilan T. L. Hauer A. (2019). Loss of MPC1 reprograms retinal metabolism to impair visual function. Proc. Natl. Acad. Sci. U. S. A. 116 (9), 3530–3535. 10.1073/pnas.1812941116 30808746 PMC6397593

[B14] Gulati G. S. Sikandar S. S. Wesche D. J. Manjunath A. Bharadwaj A. Berger M. J. (2020). Single-cell transcriptional diversity is a hallmark of developmental potential. Science 367 (6476), 405–411. 10.1126/science.aax0249 31974247 PMC7694873

[B15] Hanzelmann S. Castelo R. Guinney J. (2013). Gsva: gene set variation analysis for microarray and RNA-seq data. BMC Bioinforma. 14, 7. 10.1186/1471-2105-14-7 PMC3618321 23323831

[B16] Hazra A. Gogtay N. (2016). Biostatistics series module 3: comparing groups: numerical variables. Indian J. Dermatol 61 (3), 251–260. 10.4103/0019-5154.182416 27293244 PMC4885176

[B17] Ippolito L. Morandi A. Giannoni E. Chiarugi P. (2019). Lactate: a metabolic driver in the tumour landscape. Trends Biochem. Sci. 44 (2), 153–166. 10.1016/j.tibs.2018.10.011 30473428

[B18] Jiang H. Alahmad A. Fu S. Fu X. Liu Z. Han X. (2022). Identification and characterization of novel MPC1 gene variants causing mitochondrial pyruvate carrier deficiency. J. Inherit. Metab. Dis. 45 (2), 264–277. 10.1002/jimd.12462 34873722

[B19] Jin Z. Lu Y. Wu X. Pan T. Yu Z. Hou J. (2021). The cross-talk between tumor cells and activated fibroblasts mediated by lactate/BDNF/TrkB signaling promotes acquired resistance to anlotinib in human gastric cancer. Redox Biol. 46, 102076. 10.1016/j.redox.2021.102076 34315112 PMC8326414

[B20] Joyce J. A. Fearon D. T. (2015). T cell exclusion, immune privilege, and the tumor microenvironment. Science 348 (6230), 74–80. 10.1126/science.aaa6204 25838376

[B21] Koppenol W. H. Bounds P. L. Dang C. V. (2011). Otto Warburg's contributions to current concepts of cancer metabolism. Nat. Rev. Cancer 11 (5), 325–337. 10.1038/nrc3038 21508971

[B22] Kraehenbuehl L. Weng C. H. Eghbali S. Wolchok J. D. Merghoub T. (2022). Enhancing immunotherapy in cancer by targeting emerging immunomodulatory pathways. Nat. Rev. Clin. Oncol. 19 (1), 37–50. 10.1038/s41571-021-00552-7 34580473

[B23] Kumagai S. Koyama S. Itahashi K. Tanegashima T. Lin Y. T. Togashi Y. (2022). Lactic acid promotes PD-1 expression in regulatory T cells in highly glycolytic tumor microenvironments. Cancer Cell. 40 (2), 201–218 e9. 10.1016/j.ccell.2022.01.001 35090594

[B24] Lech G. Słotwiński R. Słodkowski M. Krasnodębski I. W. (2016). Colorectal cancer tumour markers and biomarkers: recent therapeutic advances. World J. Gastroenterol. 22 (5), 1745–1755. 10.3748/wjg.v22.i5.1745 26855534 PMC4724606

[B25] Lee H. O. Hong Y. Etlioglu H. E. Cho Y. B. Pomella V. Van den Bosch B. (2020). Lineage-dependent gene expression programs influence the immune landscape of colorectal cancer. Nat. Genet. 52 (6), 594–603. 10.1038/s41588-020-0636-z 32451460

[B26] Lian X. Yang K. Li R. Li M. Zuo J. Zheng B. (2022). Immunometabolic rewiring in tumorigenesis and anti-tumor immunotherapy. Mol. Cancer 21 (1), 27. 10.1186/s12943-021-01486-5 35062950 PMC8780708

[B27] Madden M. Z. Rathmell J. C. (2021). The complex integration of T-cell metabolism and immunotherapy. Cancer Discov. 11 (7), 1636–1643. 10.1158/2159-8290.CD-20-0569 33795235 PMC8295173

[B28] Marisa L. de Reyniès A. Duval A. Selves J. Gaub M. P. Vescovo L. (2013). Gene expression classification of colon cancer into molecular subtypes: characterization, validation, and prognostic value. PLoS Med. 10 (5), e1001453. 10.1371/journal.pmed.1001453 23700391 PMC3660251

[B29] Muller C. Schillert A. Röthemeier C. Trégouët D. A. Proust C. Binder H. (2016). Removing batch effects from longitudinal gene expression - quantile normalization plus ComBat as best approach for microarray transcriptome data. PLoS One 11 (6), e0156594. 10.1371/journal.pone.0156594 27272489 PMC4896498

[B30] Newman A. M. Liu C. L. Green M. R. Gentles A. J. Feng W. Xu Y. (2015). Robust enumeration of cell subsets from tissue expression profiles. Nat. Methods 12 (5), 453–457. 10.1038/nmeth.3337 25822800 PMC4739640

[B31] Quail D. F. Joyce J. A. (2013). Microenvironmental regulation of tumor progression and metastasis. Nat. Med. 19 (11), 1423–1437. 10.1038/nm.3394 24202395 PMC3954707

[B32] Rabanal-Ruiz Y. Llanos-Gonzalez E. Alcain F. J. (2021). The use of coenzyme Q10 in cardiovascular diseases. Antioxidants (Basel) 10 (5), 755. 10.3390/antiox10050755 34068578 PMC8151454

[B33] Satija R. Farrell J. A. Gennert D. Schier A. F. Regev A. (2015). Spatial reconstruction of single-cell gene expression data. Nat. Biotechnol. 33 (5), 495–502. 10.1038/nbt.3192 25867923 PMC4430369

[B34] Shang M. Cappellesso F. Amorim R. Serneels J. Virga F. Eelen G. (2020). Macrophage-derived glutamine boosts satellite cells and muscle regeneration. Nature 587 (7835), 626–631. 10.1038/s41586-020-2857-9 33116312 PMC7116844

[B35] Sung H. Ferlay J. Siegel R. L. Laversanne M. Soerjomataram I. Jemal A. (2021). Global cancer statistics 2020: gLOBOCAN estimates of incidence and mortality worldwide for 36 cancers in 185 countries. CA Cancer J. Clin. 71 (3), 209–249. 10.3322/caac.21660 33538338

[B36] Szeglin B. C. Wu C. Marco M. R. Park H. S. Zhang Z. Zhang B. (2022). A SMAD4-modulated gene profile predicts disease-free survival in stage II and III colorectal cancer. Cancer Rep. Hob. 5 (1), e1423. 10.1002/cnr2.1423 PMC8789617 34114372

[B37] Vaupel P. Schmidberger H. Mayer A. (2019). The warburg effect: essential part of metabolic reprogramming and central contributor to cancer progression. Int. J. Radiat. Biol. 95 (7), 912–919. 10.1080/09553002.2019.1589653 30822194

[B38] Wang J. X. Choi S. Y. C. Niu X. Kang N. Xue H. Killam J. (2020). Lactic acid and an acidic tumor microenvironment suppress anticancer immunity. Int. J. Mol. Sci. 21 (21), 8363. 10.3390/ijms21218363 33171818 PMC7664620

[B39] Wilkerson M. D. Hayes D. N. (2010). ConsensusClusterPlus: a class discovery tool with confidence assessments and item tracking. Bioinformatics 26 (12), 1572–1573. 10.1093/bioinformatics/btq170 20427518 PMC2881355

[B40] Xia L. Oyang L. Lin J. Tan S. Han Y. Wu N. (2021). The cancer metabolic reprogramming and immune response. Mol. Cancer 20 (1), 28. 10.1186/s12943-021-01316-8 33546704 PMC7863491

[B41] Zeng D. Li M. Zhou R. Zhang J. Sun H. Shi M. (2019). Tumor microenvironment characterization in gastric cancer identifies prognostic and immunotherapeutically relevant gene signatures. Cancer Immunol. Res. 7 (5), 737–750. 10.1158/2326-6066.CIR-18-0436 30842092

[B42] Zhang Y. Zhang Z. (2020). The history and advances in cancer immunotherapy: understanding the characteristics of tumor-infiltrating immune cells and their therapeutic implications. Cell. Mol. Immunol. 17 (8), 807–821. 10.1038/s41423-020-0488-6 32612154 PMC7395159

