# Supplementary figures and images for: Exploring the relationship between lactate metabolism and immunological function in colorectal cancer through genes identification and analysis
Source: Front Cell Dev Biol. 2023 Aug 24;11:1173803. doi: 10.3389/fcell.2023.1173803 (PMC10484590; doi:10.3389/fcell.2023.1173803)

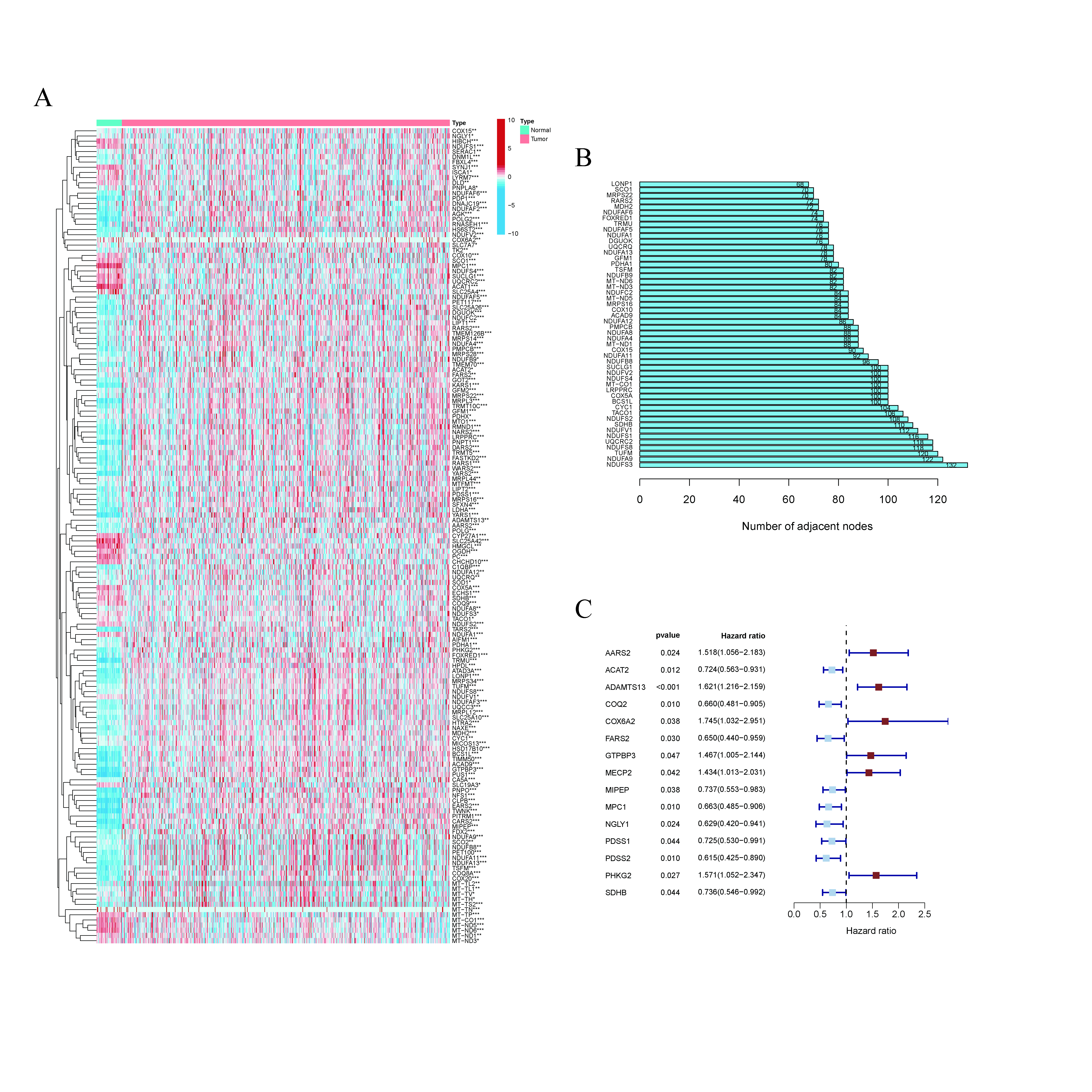

Supplement: Supplementary file 1 [file DataSheet1.ZIP › Addition Files/Supplementary Figure S1.jpg]

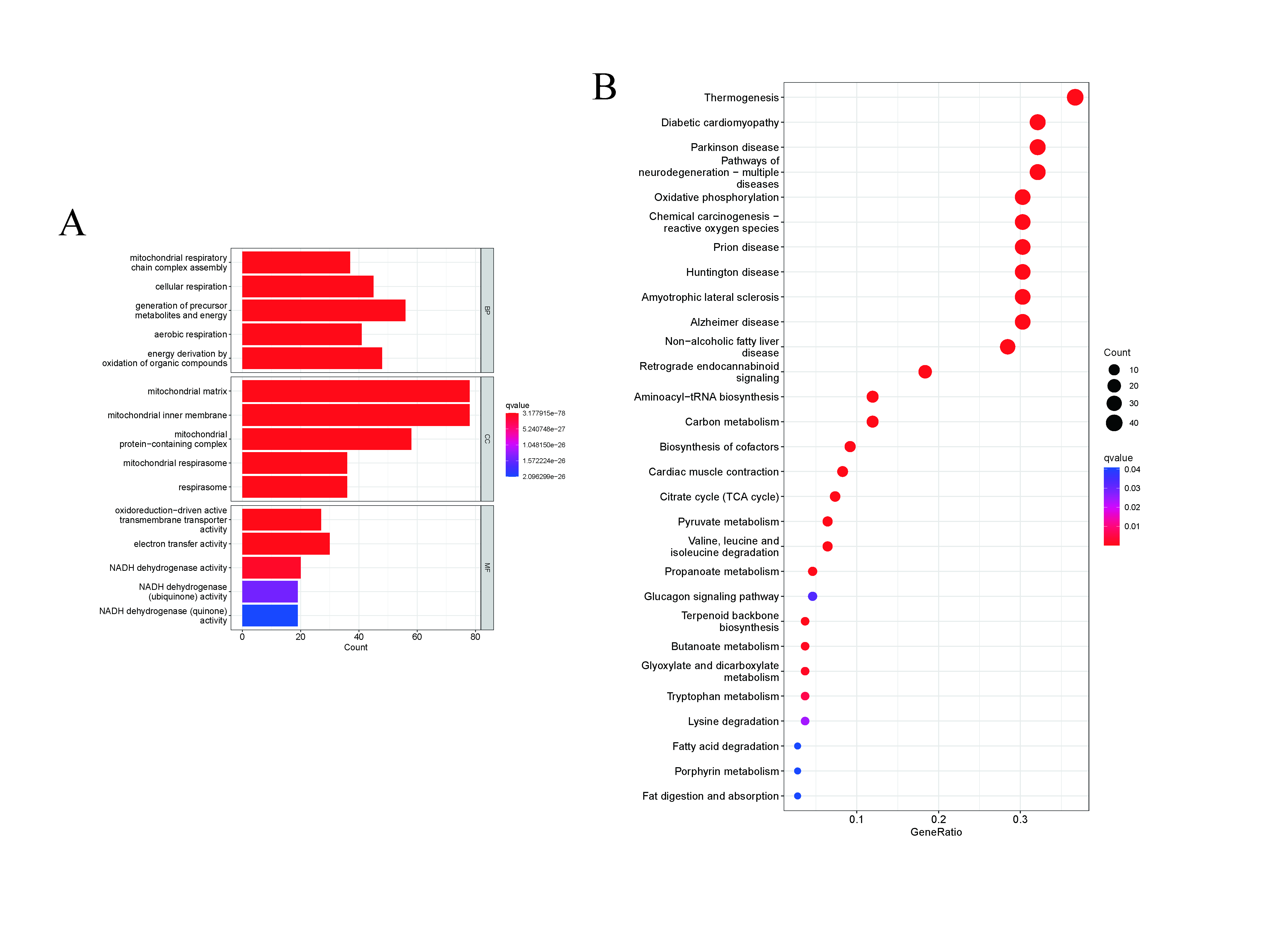

Supplement: Supplementary file 1 [file DataSheet1.ZIP › Addition Files/Supplementary Figure S2.jpg]

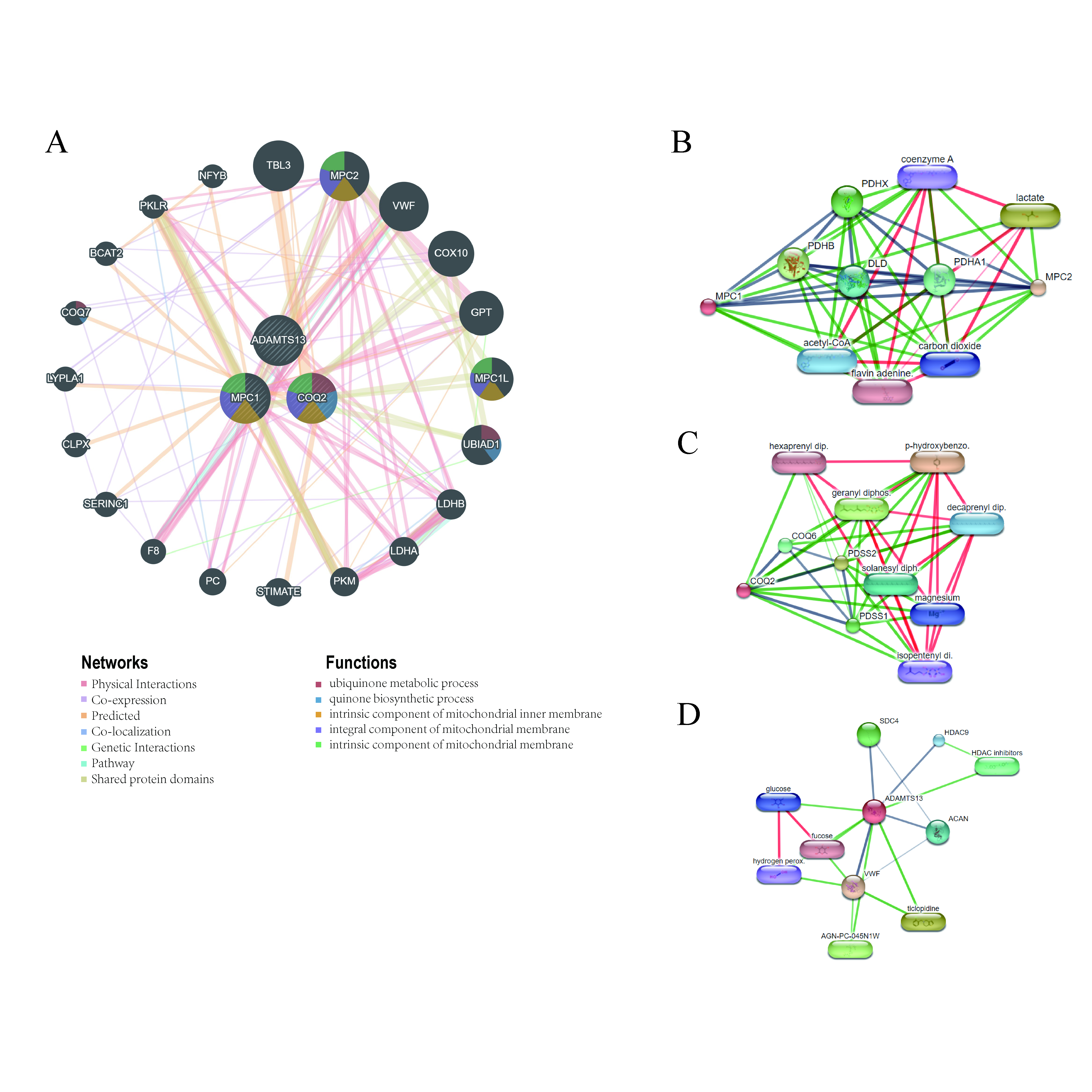

Supplement: Supplementary file 1 [file DataSheet1.ZIP › Addition Files/Supplementary Figure S3.jpg]

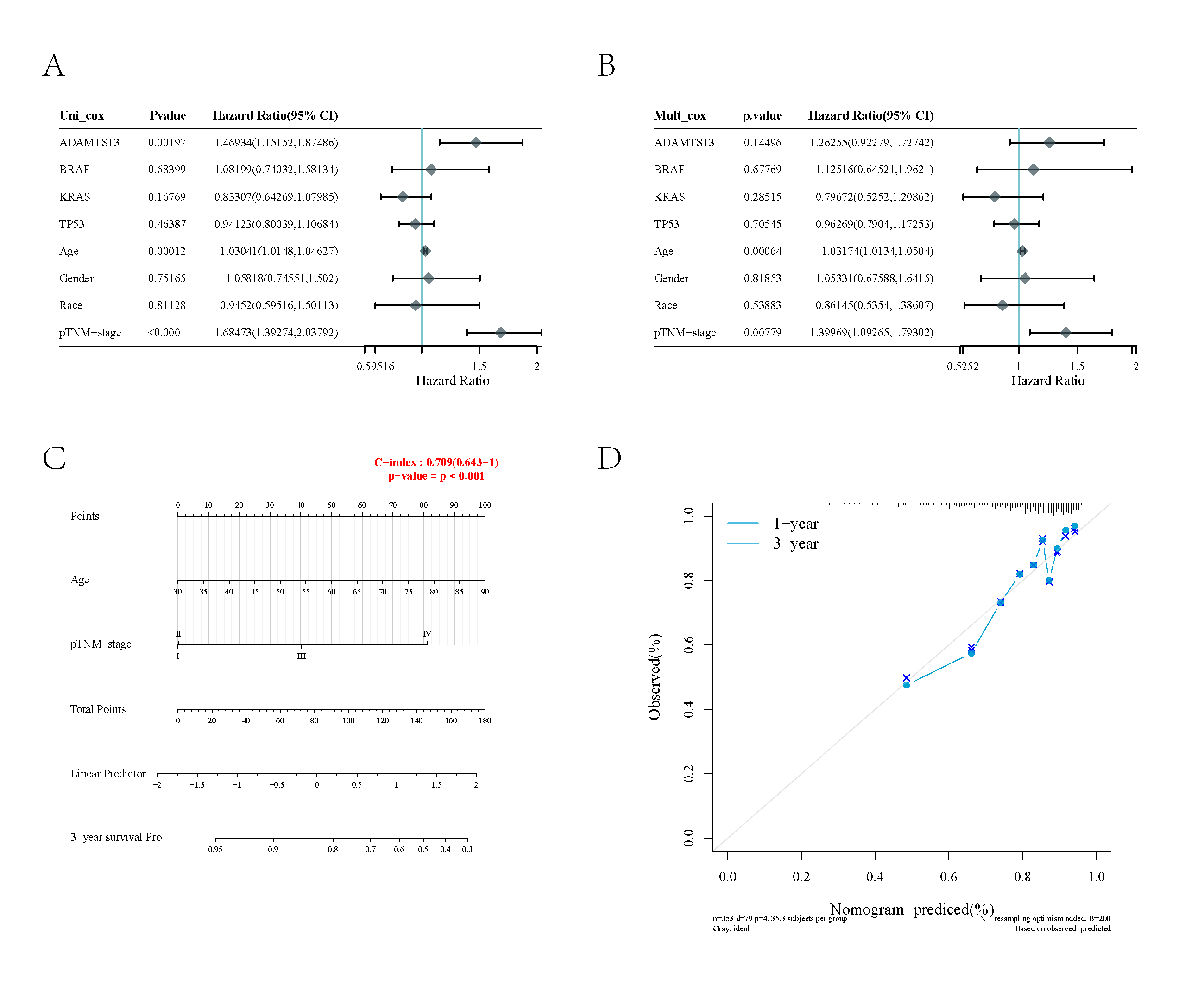

Supplement: Supplementary file 1 [file DataSheet1.ZIP › Addition Files/Supplementary Figure S4.jpg]

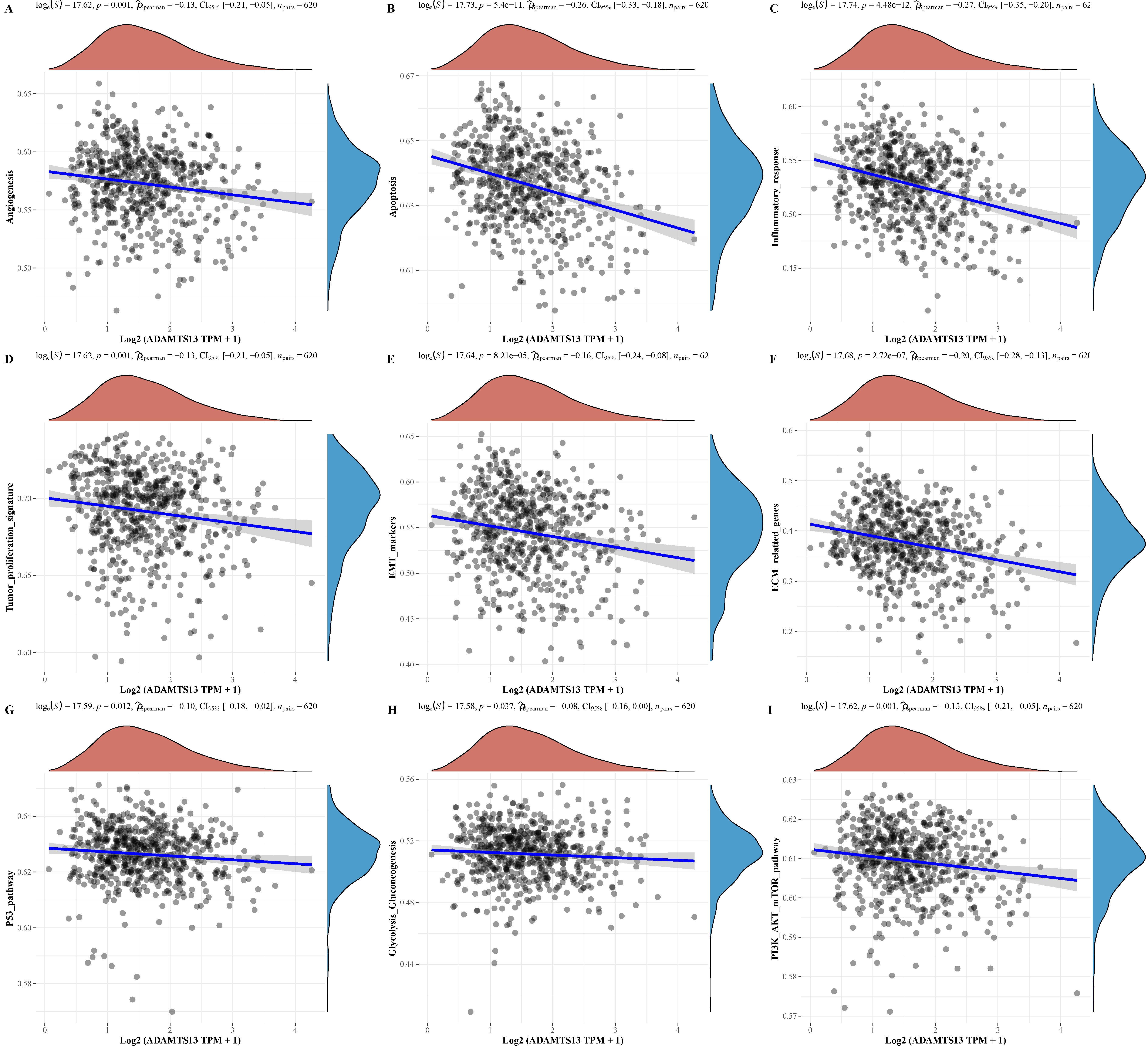

Supplement: Supplementary file 1 [file DataSheet1.ZIP › Addition Files/Supplementary Figure S5.jpg]

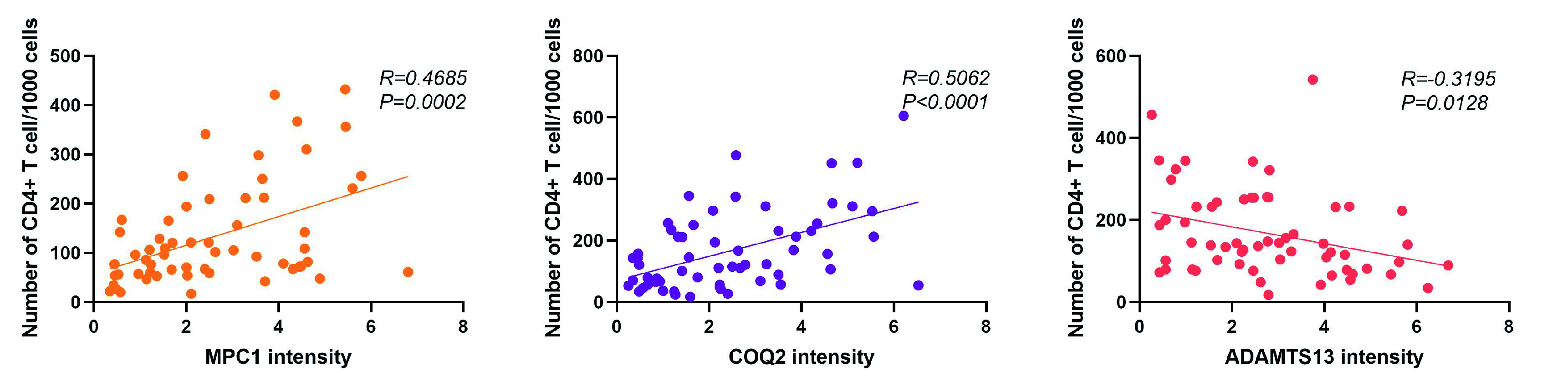

Supplement: Supplementary file 1 [file DataSheet1.ZIP › Addition Files/Supplementary Figure S6.jpg]
